# A Case of Traumatic Tetanus, Treated with Hyd. Chloral

**Published:** 1870-12

**Authors:** A. H. Kinnear

**Affiliations:** Metamora, Ill.


ARTICLE II.—*A Case of Traumatic Tetanus, treated with Hyd. Chloral.* By A. H. KINNEAR, M.D., Metamora, Ill.

On Monday, Sept. 19th, 1870, a summons came to my office requesting my services some three miles east of town, to see a little German boy, eight years old. I was not at home when the messenger arrived, who stated that his orders were, if I was not at home, to have my student, Mr. F. G. Roberts, visit the boy until I returned. Mr. R. rather reluctantly answered the summons, and found the little boy strangely affected. He did not wish to administer any medicine until I could see the case, and got permission of the parents to bring the boy to town, and have him remain over night at my house, so that I might see him that evening, as it was nearly sundown when the summons came. Shortly after the boy was brought to my house I came home, and was not a little surprised to find a marked case of tetanus. The little fellow was suffering terribly from the persistent rigidity of the muscles of the lower jaw, the neck, and of the trunk. Opisthotonic spasms at intervals of from half to one hour. During these spasms, the whole body would be bent backward in the form of an arch, resting upon the occiput and sacrum. Trismus persistent; could not open his mouth more than half an inch; deglutition slow and difficult; pulse normal, except during the paroxysms, when they would become a little accelerated; no trouble in voiding urine.

I learned the history of the case to be as follows:

On Friday night, the 16th, this little boy fell out of the bed on the floor, striking on the back of his head and spine, but got up and went to bed again, not complaining of its hurting him, at the time, further than a stunning sensation; slept well the remainder of the night. The next morning, the 17th, the parents noticed that he acted strangely—also, an unnatural expression of the features, but gave themselves no uneasiness until late that afternoon, when they noticed that he could not articulate distinctly, mastication and deglutition rather difficult, and the muscles of the jaw becoming stiff.

During the night he slept but little, consequent upon pain and occasionally a light spasm. The next morning, the 18th, they observed that the muscles of the neck and trunk were becoming stiff, with opisthotonos at long intervals, but not very severe.

He continued in this condition, the characteristic symptoms becoming more marked, until Monday, the 19th, when some of the neighbors prevailed on them to have a physician called; the parents thinking, all the time, that his condition resulted from cold, which he would probably recover from in a short time, without the interference of a physician.

The above being the facts in the case as near as I could gather them, I diagnosed the case traumatic tetanus, as the boy was in good health at the time of the accident, and had been all his life, although I could discover no abrasion of the skin, only a little tenderness at the upper half of the cervical spine.

Being aware that recovery from this disease is the exception, and death, so to speak, the rule, I was at a loss to know what would be best, in the case, to relieve the rigid condition of the muscular system, from which the vital forces were fast being exhausted.

The authorities state that there is no remedial agent that can be relied upon in this affection. This being the state of affairs, I felt justified in experimenting in the case, and, therefore, concluded to try Hyd. Chloral. I administered fifteen grains in solution, and in twenty minutes he dropped off into a quiet sleep, and slept about one hour; then awoke suddenly in a spasm, which lasted about one minute. I then gave him twenty grains more; he went right to sleep, and slept very quietly the greater part of the night, awakening twice, partly, only, in a light spasm. The next morning, without my knowledge, he got up, and dressed himself, saying that he felt better. He could open his mouth a little wider than the evening before, but not sufficiently to take food except in a semi-liquid form. After breakfast I gave him fifteen grains more of Chloral, and had him removed to his home, with orders that he should be placed in a recumbent position, and remain perfectly quiet; nourishment to be given every four hours, the Chloral every six hours, twenty grains at each dose.

The next morning, the 21st, I found my little patient very much relieved. He had rested quietly all night; the muscles of the jaw were more relaxed, also of the neck and trunk; but those of the lower extremities were becoming a little rigid. Had only four attacks of opisthotonos since he left my house, which were not so severe, and shorter in duration. I gave him one ounce of Castor Oil and five grains of Calomel, and continued the Chloral in the same doses as before, at intervals of six hours, or oftener, if it became necessary.

The 22nd, my patient rested well through the night; the calomel and oil had produced copious evacuation from the bowels; the muscles of the jaw, neck and trunk still more relaxed; he could eat a little this morning. Had no spasm since I last saw him, but the lower extremities more affected; continued the Chloral and nourishment the same as at last visit.

23rd. Still improving; nearly relieved of the trismus; rested tolerably well through the night; had two opisthotonic seizures since my last visit, one of which occurred during the night. The rigidity of the muscles of the lower extremities, however, no better, and during the spasms the penis would become erect with contraction of the scrotum and drawing up of the testicles, so much so as to cause great pain in the parts; the bowels moved freely twice. Continued the Chloral in the same quantity at intervals of eight hours, and added Bromide of Potassium in solution, twenty grains at each dose, to alternate with the Chloral; nourishment to be increased in quantity.

24th. Gradually improving in every particular. But one spasm since my last visit, caused by imprudence. The scrotum remaining in a contracted condition, but causing no pain; considerable stiffness in the muscles of the thighs, but not quite so rigid; the pulse more frequent than at any time previous, in consequence of exhaustion; respiration natural; no difficulty in voiding urine. Continued treatment the same as at last visit.

25th. Improvement more marked in general appearance than at any time previous, trismus having nearly or quite subsided; can articulate very distinctly; takes food as readily as before being affected; had two very light spasms, one while I was there, which seemed to affect the muscles of the thighs more than the trunk, and as soon as this passed off, the whole muscular system became relaxed, except the muscles of the thighs.

I now began to feel very much encouraged about my patient's recovery, as this was the ninth day since the disease first began to manifest itself. Having succeeded in relieving almost entirely the trismus, the rigidity of the muscles of the neck and trunk, the only remaining difficulty now was to relieve the stiffness of the muscles of the lower extremities, and tone up more thoroughly the whole system, which had become very much reduced from the long continued contraction of all the voluntary muscles. I continued the chloral and bromide in less quantity, but at shorter intervals; nourishment to be increased in quantity; and decided that at my next visit, if the contractiou of the muscles of the thighs continued, I would try the effect of morphia (sulph.) hypodermically, and the electric current. Left my patient, expecting to see him the next day.

26th. On my way out this morning to visit my patient, I met the father of the boy, who informed me that through the influence of a brother-in-law, who had called in to see the boy for the first time since his illness, he had been persuaded to call another physician of this place, of greater experience, to take charge of the case, without informing me. The physician in question pronounced it *rheumatism*, and treated it, I presume, accordingly. Seven days more elapsed, and the boy was a corpse.

This is rather an incomplete case to report, but as I took notes in full from the start, with the object of reporting it in the JOURNAL, I feel desirous yet to do so, as far as I was connected with the case, to show the influence that the hyd. chloral has in ameliorating the rigid condition of the muscles in this affection. I am inclined to believe that this is accomplished more through its hypnotic influence than that of any other. Its effects were beyond my expectation. The bromide, probably, had some effect in allaying nervousness, but to what extent, in this terrible affection, I am not able to say. There seems to be no danger in keeping the system thoroughly under the influence of chloral, any reasonable length of time, and by this means we succeed in procuring rest and quiet sleep for our patient, which has a marked tendency to interrupt the tonic spasm of the muscles; and I have great reason to believe that the treatment adopted in this case, if it had been followed, with plenty of nourishment, would have been successful, and that the patient would have recovered.

